# SVD-phy: improved prediction of protein functional associations through singular value decomposition of phylogenetic profiles

**DOI:** 10.1093/bioinformatics/btv696

**Published:** 2015-11-26

**Authors:** Andrea Franceschini, Jianyi Lin, Christian von Mering, Lars Juhl Jensen

**Affiliations:** ^1^; ^2^; ^3^; ^4^

## Abstract

**Summary:** A successful approach for predicting functional associations between non-homologous genes is to compare their phylogenetic distributions. We have devised a phylogenetic profiling algorithm, SVD-Phy, which uses truncated singular value decomposition to address the problem of uninformative profiles giving rise to false positive predictions. Benchmarking the algorithm against the KEGG pathway database, we found that it has substantially improved performance over existing phylogenetic profiling methods.

**Availability and implementation:** The software is available under the open-source BSD license at https://bitbucket.org/andrea/svd-phy

**Contact:** lars.juhl.jensen@cpr.ku.dk

**Supplementary information:** Supplementary data are available at *Bioinformatics* online.

## 1 Introduction

Phylogenetic profiling methods are able to predict functional interactions between genes that encode proteins from the same complex or pathway, by comparing their phylogenetic distributions (Cheng and Perocchi, 2015; Date and Marcotte, 2003; Enault *et al.*, 2003; Li *et al.*, 2014; Pellegrini *et al.*, 1999; Tabach *et al.*, 2013a). The underlying idea is that when two genes are functionally related, they should tend to be co-inherited; since the loss of either one of these genes would be detrimental to that particular function. Here we present a new phylogenetic profiling method, SVD-Phy, which performs considerably better than existing methods for both bacteria and eukaryotes.

## 2 Phylogenetic profiling algorithm

Our algorithm infers associations among the proteins in a query organism based on their sequence similarity to sequences from a large number of other organisms. Specifically we construct a matrix with the alignment *bit scores* of the best scoring match for each query protein (rows) in each organism (columns), including the organism itself. We obtain the bit scores from SIMAP (Arnold *et al.*, 2014) via the homology table of STRING v10 (Szklarczyk *et al.*, 2015), but bit scores from BLAST can also be used. If a query protein gives no hits in a certain organism with a bit score of at least 60, the bit score is set to 0; using higher cutoffs reduced the performance (Supplementary Fig. S1). We convert this matrix to a *normalized best hit* matrix *M* by dividing each bit score by the largest score in the same row (typically the self-hit).

Similar to earlier work on phylogenetic stratification (Psomopoulos *et al.*, 2013), we then perform truncated singular value decomposition (SVD) of *M* by calculating the factorization *M=USV'* and retaining only the first *C* columns from the resulting unitary matrix *U*. Different values of *C* were tested for each organism (Supplementary Figs S2–S5). We finally normalize each row in the matrix to unit vectors and calculate all pairwise Euclidean distances between them. Other similarity metrics gave similar or worse performance (Supplementary Figs S6–S10). See supplementary material for further details.

## 3 Benchmarking and comparison

We tested the algorithm on both prokaryotic and eukaryotic proteins and compared its performance against a simplified algorithm lacking the truncated SVD step and against two established algorithms (Date and Marcotte, 2003; Tabach *et al*., 2013a,b). For all four algorithms, we generated ranked lists of predicted associations based on phylogenetic profiles across all 1793 prokaryotes and 238 eukaryotes in STRING v10 for prokaryotic and eukaryotic query proteins, respectively. We benchmarked the predicted associations against the KEGG pathway database (Kanehisa *et al.*, 2014).

Given a ranked list of predicted function associations, we evaluate the performance as follows. We first discard all pairs with bit score ≥60, as homologous proteins will trivially have similar phylogenetic profiles and are often involved in the same KEGG pathway. We next map all proteins to KEGG genes and discard pairs where one or both proteins cannot be placed on a KEGG map. The remaining pairs are considered *true positives* (TP) if the two proteins fall within the same KEGG map and otherwise *false positives* (FP). To ensure that the results were not biased by certain atypical KEGG maps (Supplementary Table S1), we repeated all analysis excluding these maps. We also benchmarked the predicted associations for *E.coli* and *H.sapiens* using EcoCyc (Keseler *et al.*, 2013) and Reactome (Croft *et al.*, 2014), respectively.

**Fig. 1:**
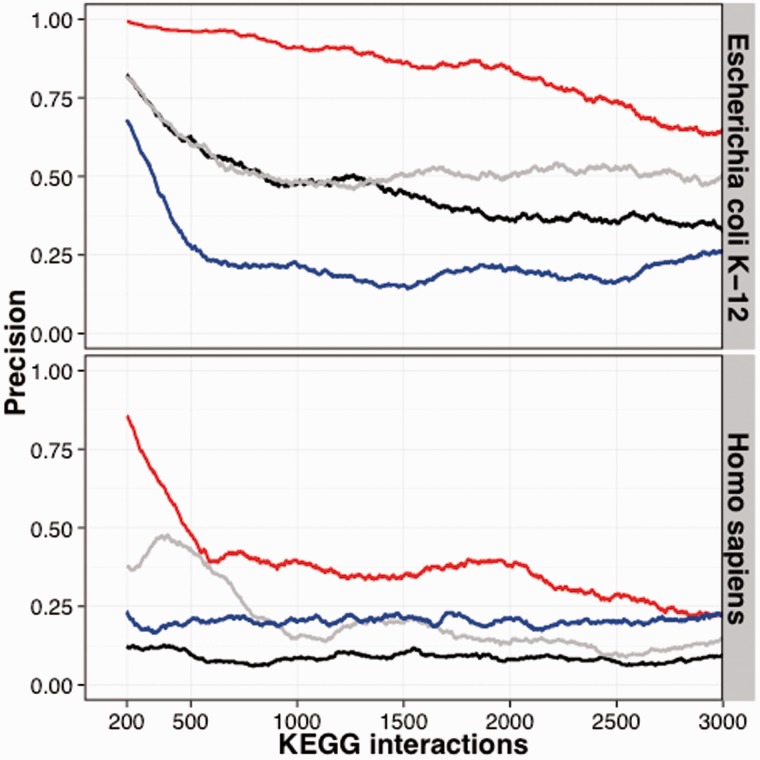
. Performance comparison of SVD-Phy and three other methods. We ran SVD-Phy (red), SVD-Phy without the truncated SVD step (gray), the Marcotte (Date and Marcotte, 2003) (black) and the Tabach (Tabach *et al.*, 2013a,b) (blue) algorithms. Graphs show the precision [TP/(TP+FP)], which we measured by scanning the sorted lists with a sliding window of 400 interactions

In all benchmarks, SVD-Phy showed dramatically improved performance over the other three algorithms, including the simplified algorithm that differs only by leaving out the truncated SVD step (Fig. 1 and Supplementary Figs S6–S10). When benchmarked on *Saccharomyces cerevisiae*, *SVD*-Phy also outperformed the CLIME method (Li *et al.*, 2014) (Supplementary Fig S11). For example, SVD-Phy predicts over 14-fold more associations at 75% precision than other methods on *Escherichia coli*, an organism on which all algorithms generally perform well. When not restricting associations to proteins that can be mapped to KEGG, we predict 14 078 interactions in *E.coli* and 4090 in *H.sapiens* at 75% precision. This corresponds to an average interaction degree of 7.2 and 0.4, respectively.

The benchmarks also revealed that all algorithms performed considerably worse on eukaryotes than on prokaryotes. To test whether this was purely due to the smaller number of eukaryotic organisms used to build the phylogenetic profiles, we repeated the analyses using profiles based on only 238 prokaryotes (Supplementary Figs S5B and S7B). Although this did lead to an expected decrease in performance, all algorithms continued to perform notably better on prokaryotes than on eukaryotes.

## 4 Discussion

We have shown that SVD-Phy has better predictive power than existing phylogenetic profiling algorithms. This improvement was achieved by performing truncated SVD on the profiles before calculating their similarities. An intuitive explanation of this transformation is that it collapses phylogenetic profiles that are shared by many proteins into fewer dimensions (principal components). This reduces noise (Psomopoulos *et al.*, 2013) and increases the diversity of the resulting profiles, which was recently shown to be beneficial (Škunca and Dessimoz, 2015). The benefit is that it prevents high similarity scores between uninformative profiles that can be trivially explained by simple vertical inheritance of genes along the taxonomic tree, or by broad similarities in the lifestyles of the organisms. This includes highly similar profiles caused by the inclusion of multiple strains of a species, clade-specific proteins and enzymes that have been lost in most parasites (because they instead import metabolites from their hosts).

We fully integrated our protein–protein interaction predictions with the STRING database (Szklarczyk *et al.*, 2015) (Supplementary Figs S12–S13). The data can be browsed online and is freely available for download in tab-delimited format. SVD-Phy executes very fast: its run time is on average about 10–20 min per organism on a normal workstation. This allows us to execute the algorithm for all 2031 species in the STRING database, and makes it possible for others to utilize the algorithm within their web resources.

In a recent study, Tabach *et al*. (2013b) successfully used their method to shed light on several disease pathways. Phylogenetic profiling algorithms have also been applied to analyze non-coding elements (NCEs), such as small RNAs (Ott *et al.*, 2012; Tabach *et al.*, 2013a), showing that phylogenetic profiling is indeed an important technique that can be used to shed light even on NCE functions and interactions (Dimitrieva and Bucher, 2012).

## Funding

This work was supported by the Swiss Institute of Bioinformatics and the Novo Nordisk Foundation [NNF14CC0001].

*Conflict of Interest:* none declared.

## Supplementary Material

Supplementary Data
