# Supplementary material for: SVD-phy: improved prediction of protein functional associations through singular value decomposition of phylogenetic profiles
Source: Bioinformatics. 2015 Nov 26;32(7):1085–7. doi: 10.1093/bioinformatics/btv696 (PMC4896368; doi:10.1093/bioinformatics/btv696)
Supplement: Supplementary Data [file supp_32_7_1085__index.html]

SVD-Phy: Improved prediction of protein functional associations through singular value decomposition of phylogenetic profiles — SVD-phy: improved prediction of protein functional associations through singular value decomposition of phylogenetic profiles — SVD-phy: improved prediction of protein functional associations through singular value decomposition of phylogenetic profiles — Supplementary Data 

# SVD-phy: improved prediction of protein functional associations through singular value decomposition of phylogenetic profiles

## Supplementary Data

files

- Supplementary Data - docx file
- Supplementary Data - xls file
