# Supplementary material for: SVD-phy: improved prediction of protein functional associations through singular value decomposition of phylogenetic profiles
Source: Bioinformatics. 2015 Nov 26;32(7):1085–7. doi: 10.1093/bioinformatics/btv696 (PMC4896368; doi:10.1093/bioinformatics/btv696)
Supplement: Supplementary Data [file supp_btv696_SVD-Phy-supplementary-material.docx]

# method

## Input data

We use the STRING v10 homology table in order to access the results of an all-against-all SIMAP search of the protein sequences. This dataset includes only hits with a *bit score* of at least 60 bits.

## Algorithm

To infer associations among the *n* proteins of an organism X based on their phylogenetic profiles across a set of *m* organisms, we do as follows:

1. We create a “Normalized Best Hit” matrix *M* of size *n* × *m*. In this matrix, every protein in X is represented by a row, which contains the bit score of the best hit of the protein in each of the *m* organisms. Every column in *M* thus represents an organism. If no hit can be found for the protein in a given organism (i.e. bit score < 60), we set that element in *M* to 0. We then normalize *M* by dividing each value by the largest value in the same row, which is typically the bit score of the self-hit. This first step is similar to Enault et al. (Enault et al., 2013).
2. Then we perform Singular Value Decomposition (SVD) of the matrix M, using the Lapack SVD function in R (<http://r-project.org>), thus obtaining the decomposition M=USV'. When performing SVD in R on an n × m matrix with n>m, the size of the resulting U matrix is also n × m.
3. Next we truncate the *U* matrix obtained from the SVD transformation, retaining only the first *C* columns, which correspond to the largest singular values. We specify *C* as a percentage of *m*. We benchmark the performance of SVD-Phy for several different values of *C* to identify a suitable choice for the organism in question.
4. We normalize each row vector of the truncated *U* by dividing it by its L^2^ norm. For every pair of rows *r_i_* and *r_j_*, each representing a protein, we compute their Euclidean distance$D_{ij}=\sqrt{\sum_{k} (r_{ik}-r_{jk})^{2}}$

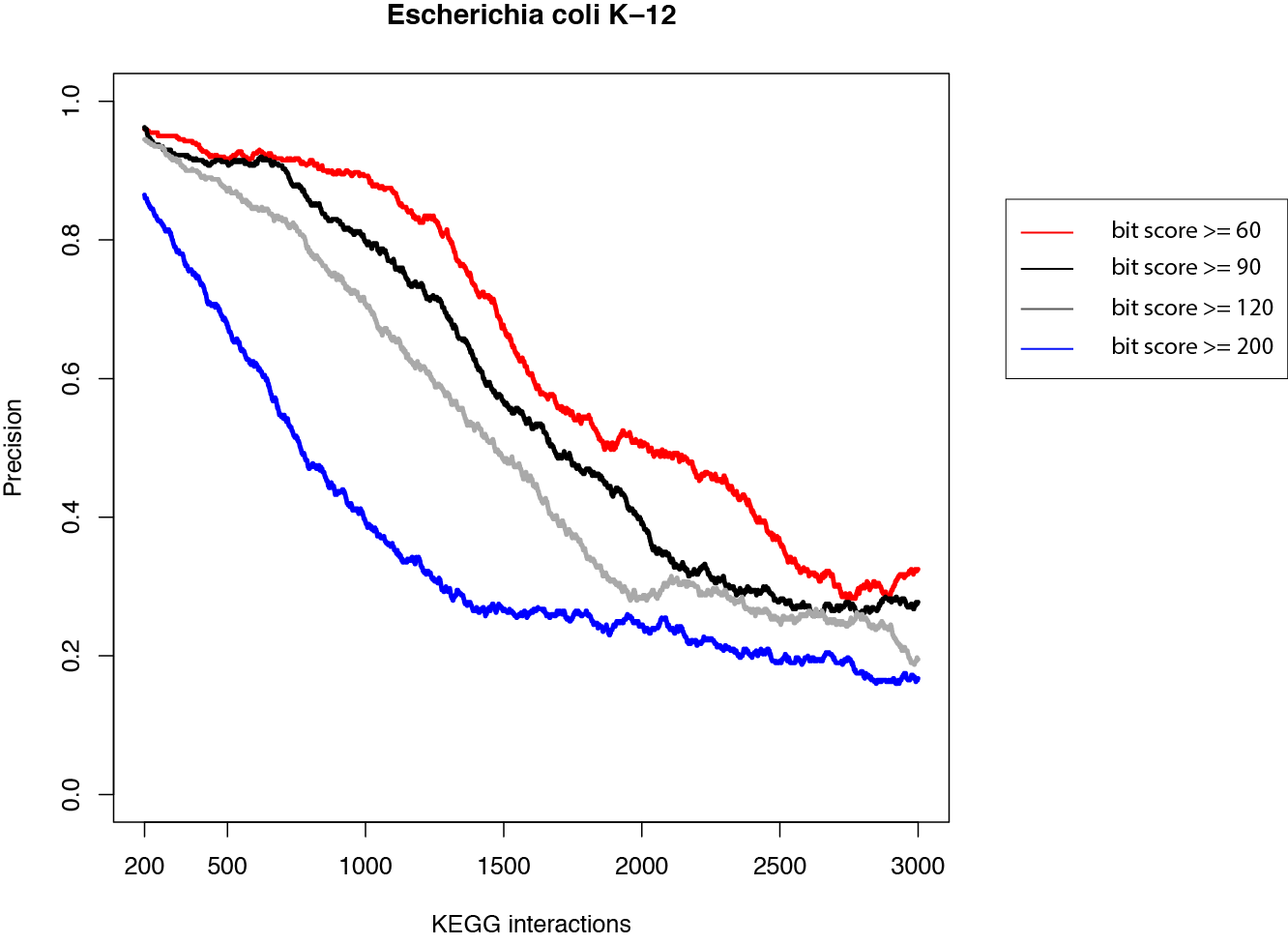


Figure S1. Impact of bit score cutoff on SVD-Phy performance. We ran SVD-Phy, applying four different thresholds on the minimum SIMAP bit score in the homology matrix, setting bit scores below the threshold to zero. Given the sorted interaction lists we mapped the proteins to KEGG genes. The graphs show the precision [TP/(TP+FP)], which we estimated by scanning the sorted lists with a sliding window of 400 interactions. The benchmark is composed of all maps available in KEGG. Increasing the bit score cutoff from 60 bits (default in SVD-Phy) to 90 bits leads to a modest decrease in performance. Even more stringent cutoffs lead to a substantial drop in performance.


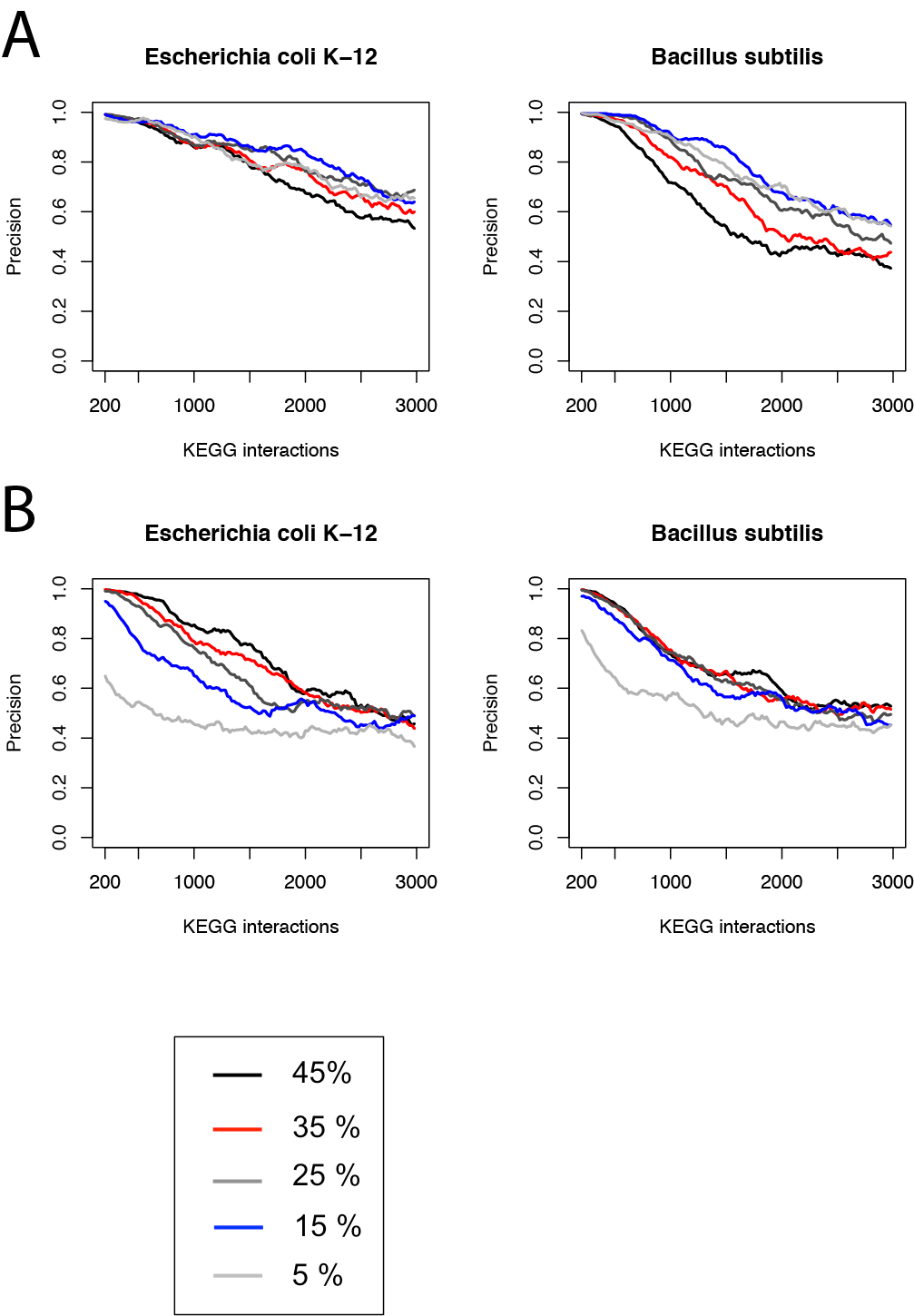


Figure S2. We executed SVD-Phy using different settings for the percentage of columns retained in the rectangular SVD matrix U. The results were computed using profiles built on *E. coli* and *B. subtilis* (A) with all the 1793 prokaryotes in STRING v10 (table S2) and (B) with a reduced set of 238 (table S3). Given the sorted interaction lists we mapped the proteins to KEGG genes. The graphs show the precision [TP/(TP+FP)], which we estimated by scanning the sorted lists with a sliding window of 400 interactions. The benchmark is composed of all maps available in KEGG.


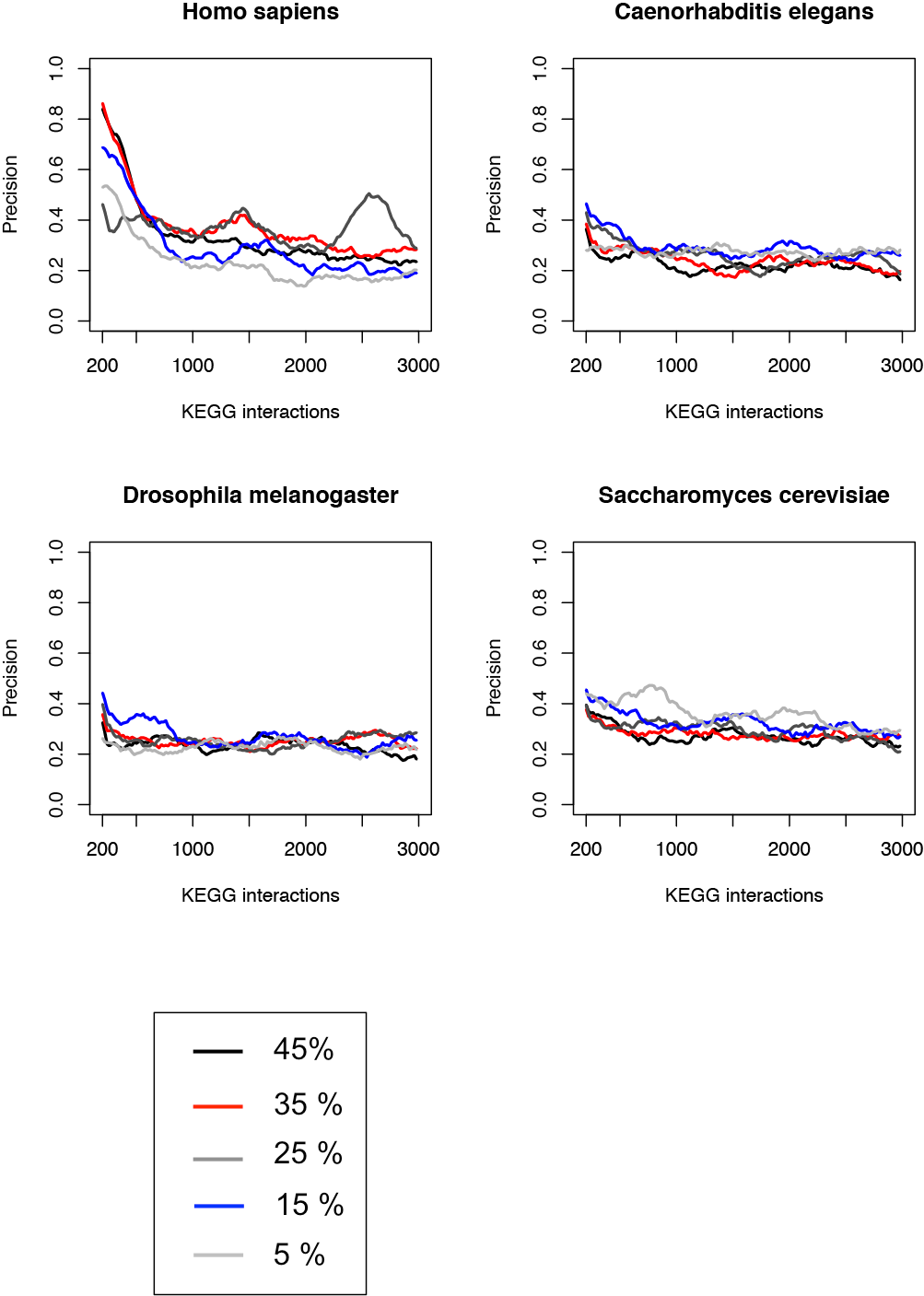


Figure S3. We executed SVD-Phy using different settings for the percentage of columns retained in the rectangular SVD matrix U. The results were computed using profiles built with all the 238 eukaryotes in STRING v10 (table S2). Given the sorted interaction lists we mapped the proteins to KEGG genes. The graphs show the precision [TP/(TP+FP)], which we estimated by scanning the sorted lists with a sliding window of 400 interactions. The benchmark is composed of all maps available in KEGG.


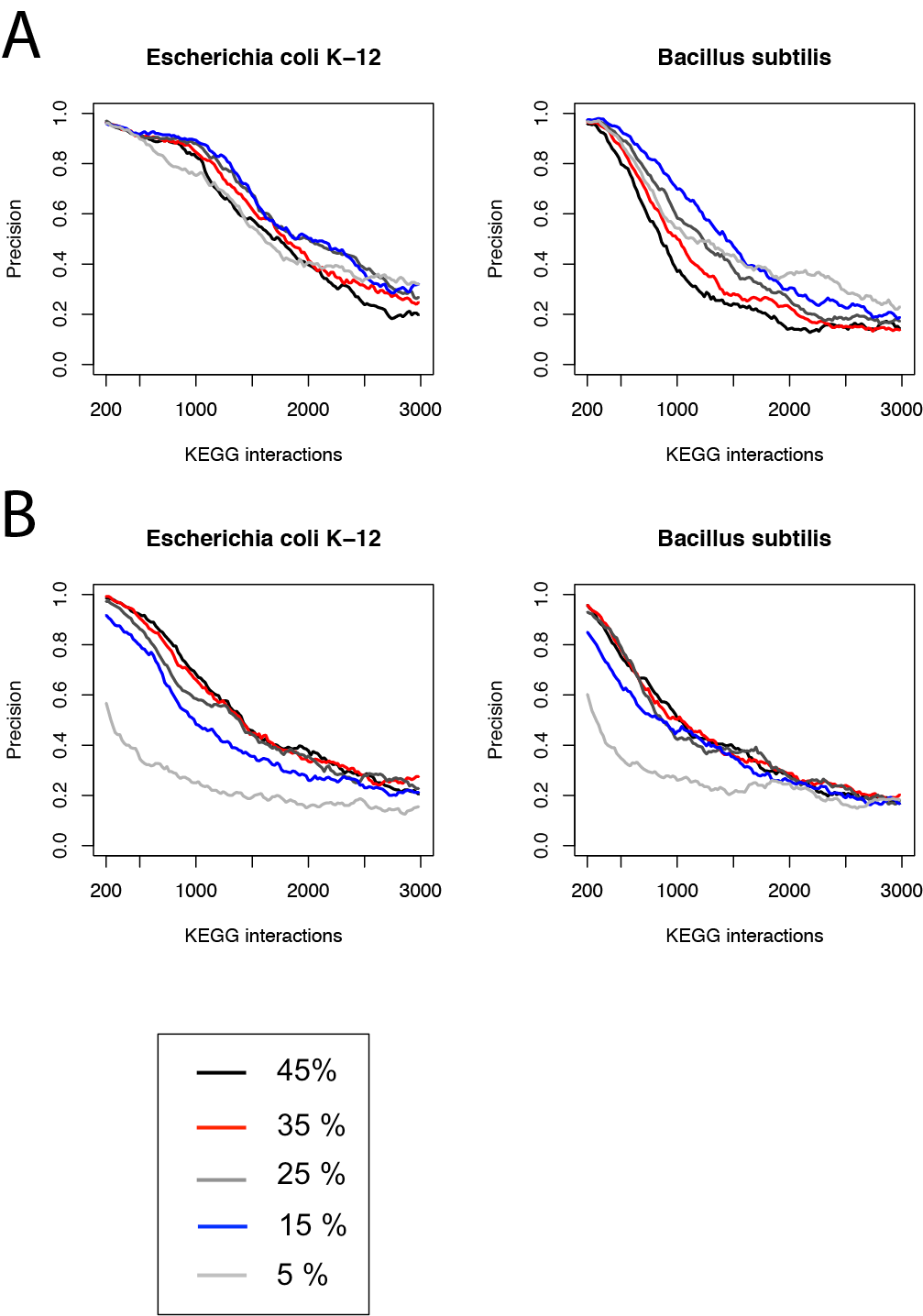


Figure S4. We executed SVD-Phy using different settings for the percentage of columns retained in the rectangular SVD matrix U. The results were computed using profiles built on *E. coli* and *B. subtilis* (A) with all the 1793 prokaryotes in STRING v10 (table S2) and (B) with a reduced set of 238 (table S3). Given the sorted interaction lists we mapped the proteins to KEGG genes. The graphs show the precision [TP/(TP+FP)], which we estimated by scanning the sorted lists with a sliding window of 400 interactions. The benchmark is composed of all maps available in KEGG except from the uninformative maps listed in table S1.


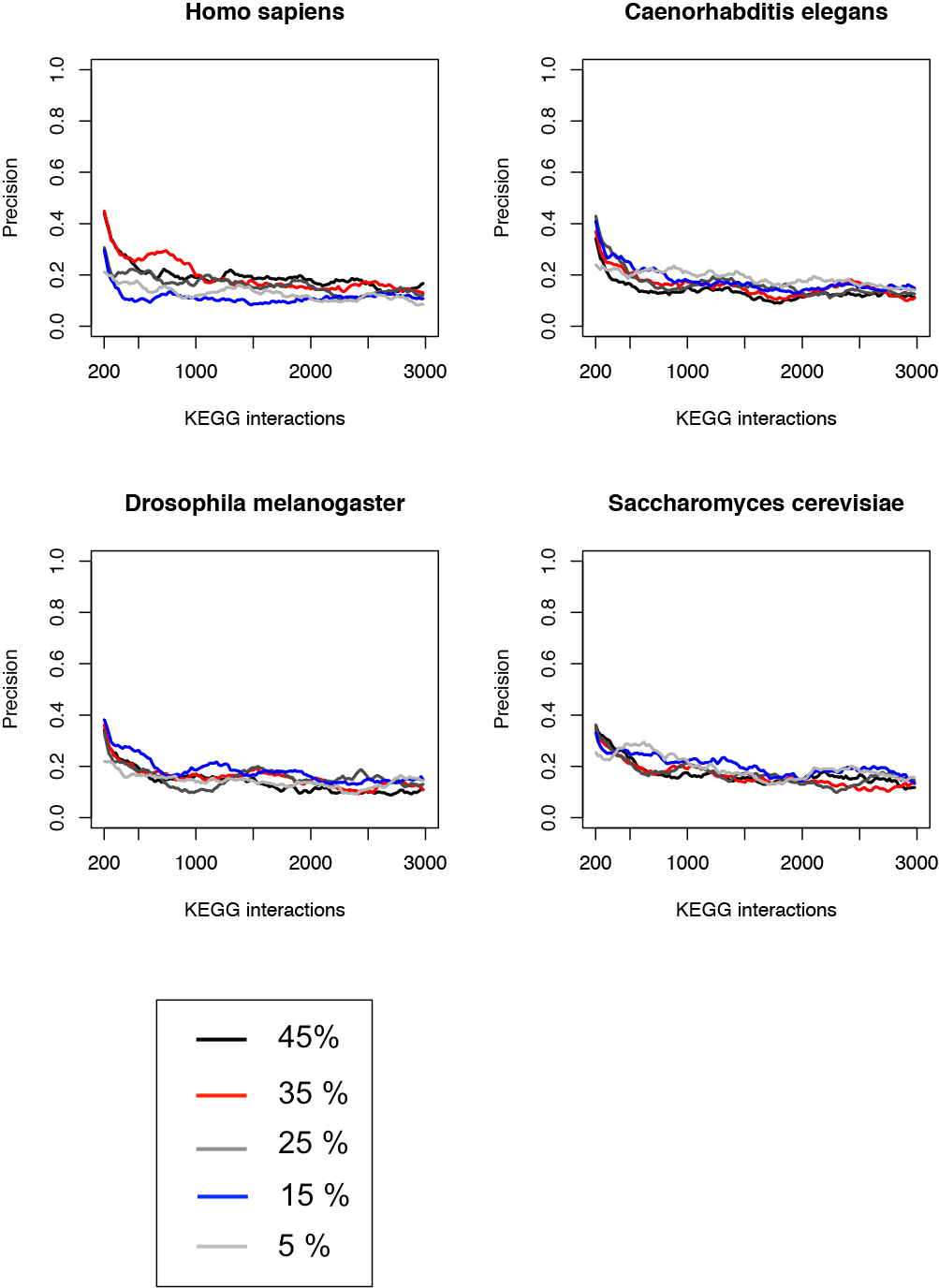


Figure S5. We executed SVD-Phy using different settings for the percentage of columns retained in the rectangular SVD matrix U. The results were computed using profiles built with all the 238 eukaryotes in STRING v10 (table S2). Given the resulted sorted interaction lists we mapped the proteins to KEGG genes. The graphs show the precision [TP/(TP+FP)], which we estimated by scanning the sorted lists with a sliding window of 400 interactions. The benchmark is composed of all maps available in KEGG except from the uninformative maps listed in table S1.


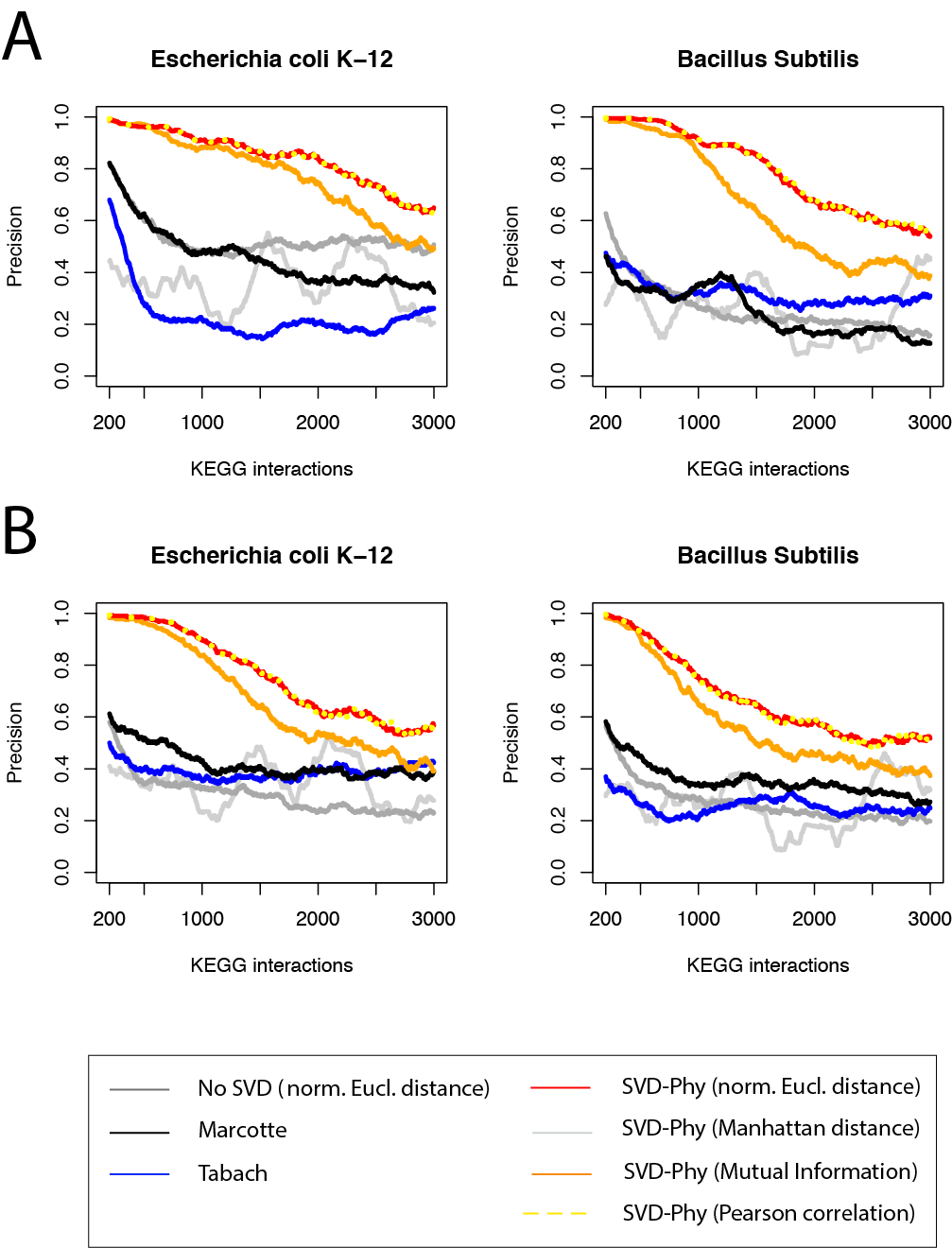


Figure S6. Benchmark of SVD-Phy, SVD-Phy using three alternative similarity metrics, SVD-Phy without the truncated SVD step, the Marcotte algorithm (Date and Marcotte, 2003) and the Tabach algorithm (Tabach et al., 2013). Graphs show the precision [TP/(TP+FP)], which we estimated by scanning the sorted lists with a sliding window of 400 interactions. We ran the algorithms using profiles composed (A) of all 1793 prokaryotes in STRING v10 (table S2), and (B) of a reduced set of 238 prokaryotes (table S3). The benchmark is composed of all the maps available in KEGG. In SVD-Phy we retained 15% of the columns from the rectangular SVD matrix U for panel A. For panel B, we retained 75% and 35% for *E. coli* and *B. subtilis*, respectively.


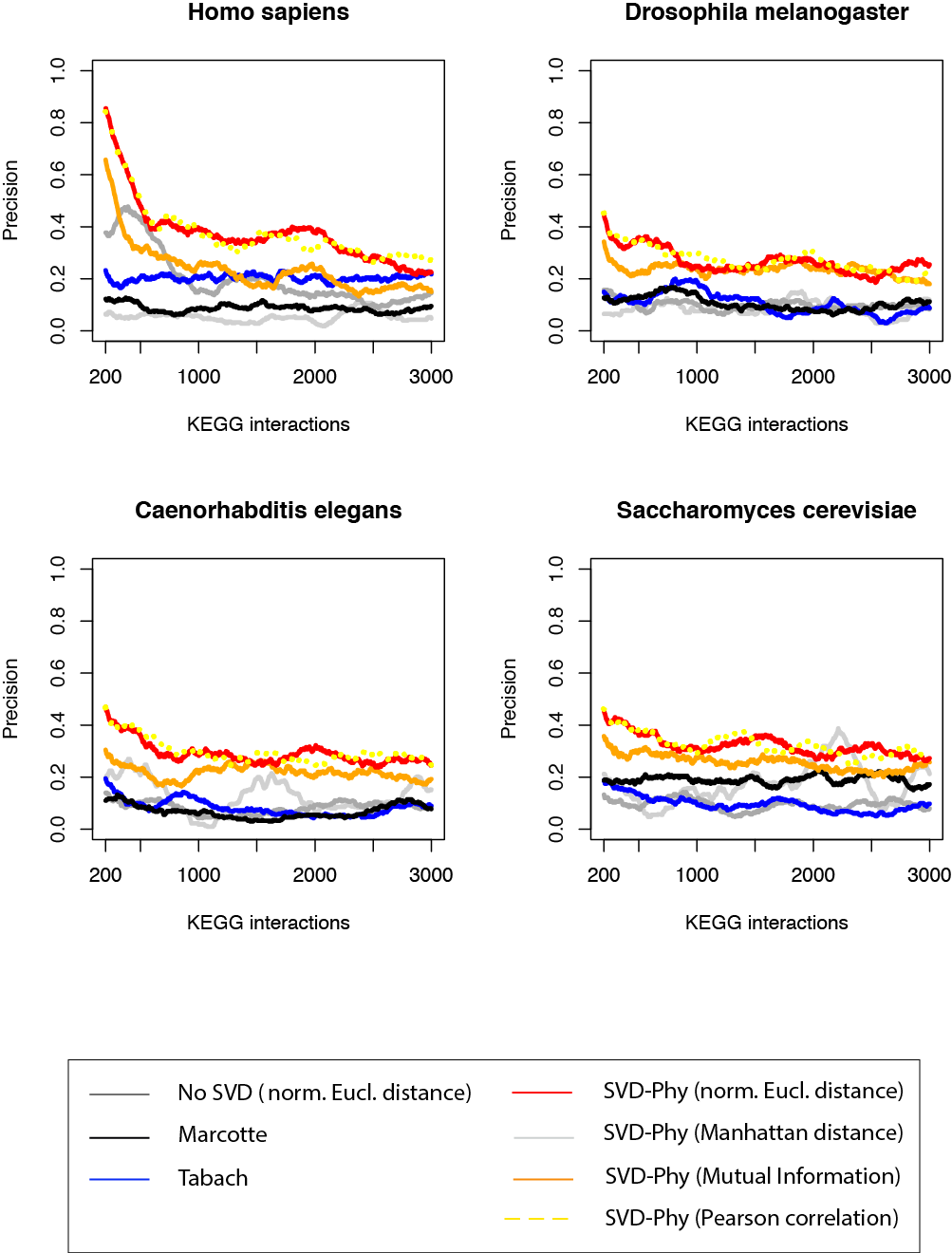


Figure S7. Benchmark of SVD-Phy, SVD-Phy using three alternative similarity metrics, SVD-Phy with the truncated SVD step disabled, the Marcotte algorithm (Date and Marcotte, 2003) and the Tabach algorithm (Tabach et al., 2013). Graphs show the precision [TP/(TP+FP)], which we estimated by scanning the sorted lists with a sliding window of 400 interactions. We ran the algorithms using profiles composed of all 238 eukaryotes in STRING v10 (table S2). The benchmark is composed of all the maps available in KEGG. In SVD-Phy we retained the following percentages of columns from the rectangular SVD matrix U: *H. sapiens* 35%, *D. melanogaster* 15%, *C. elegans* 15%, and *S. cerevisiae* 15%.


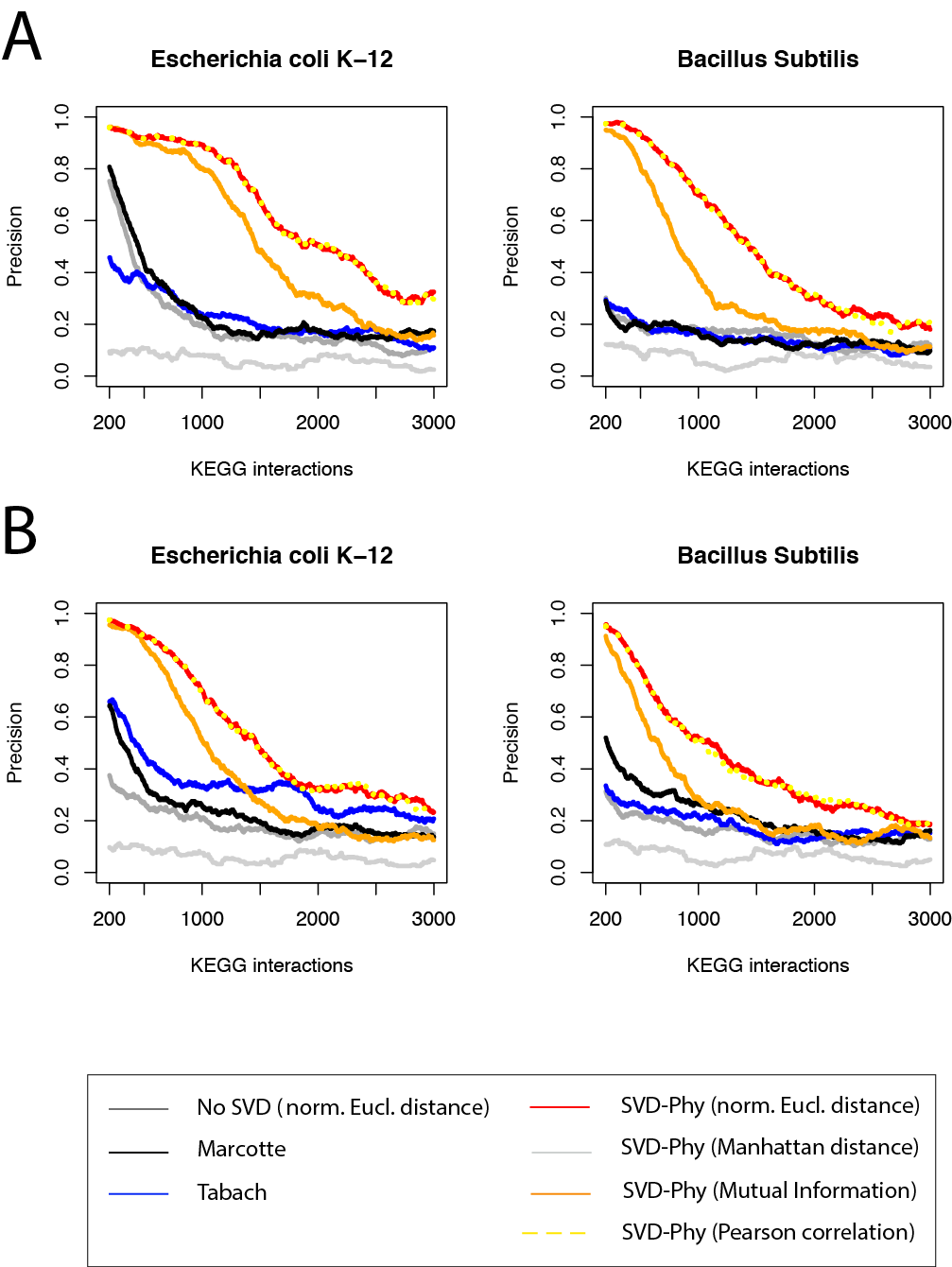


Figure S8. Benchmark of SVD-Phy, SVD-Phy using three alternative similarity metrics, SVD-Phy with the truncated SVD step disabled, the Marcotte algorithm (Date and Marcotte, 2003) and the Tabach algorithm (Tabach et al., 2013). Graphs show the precision [TP/(TP+FP)], which we estimated scanning the sorted lists with a sliding window of 400 interactions. We ran the algorithms using profiles composed (A) of all 1793 prokaryotes in STRING v10 (table S2) and (B) of a reduced set of 238 prokaryotes (table S3). We removed from KEGG the uninformative maps (table S1). In SVD-Phy we retained 15% of the columns from the rectangular SVD matrix U for panel A. For panel B, we retained 75% and 35% for *E. coli* and *B. subtilis*, respectively.


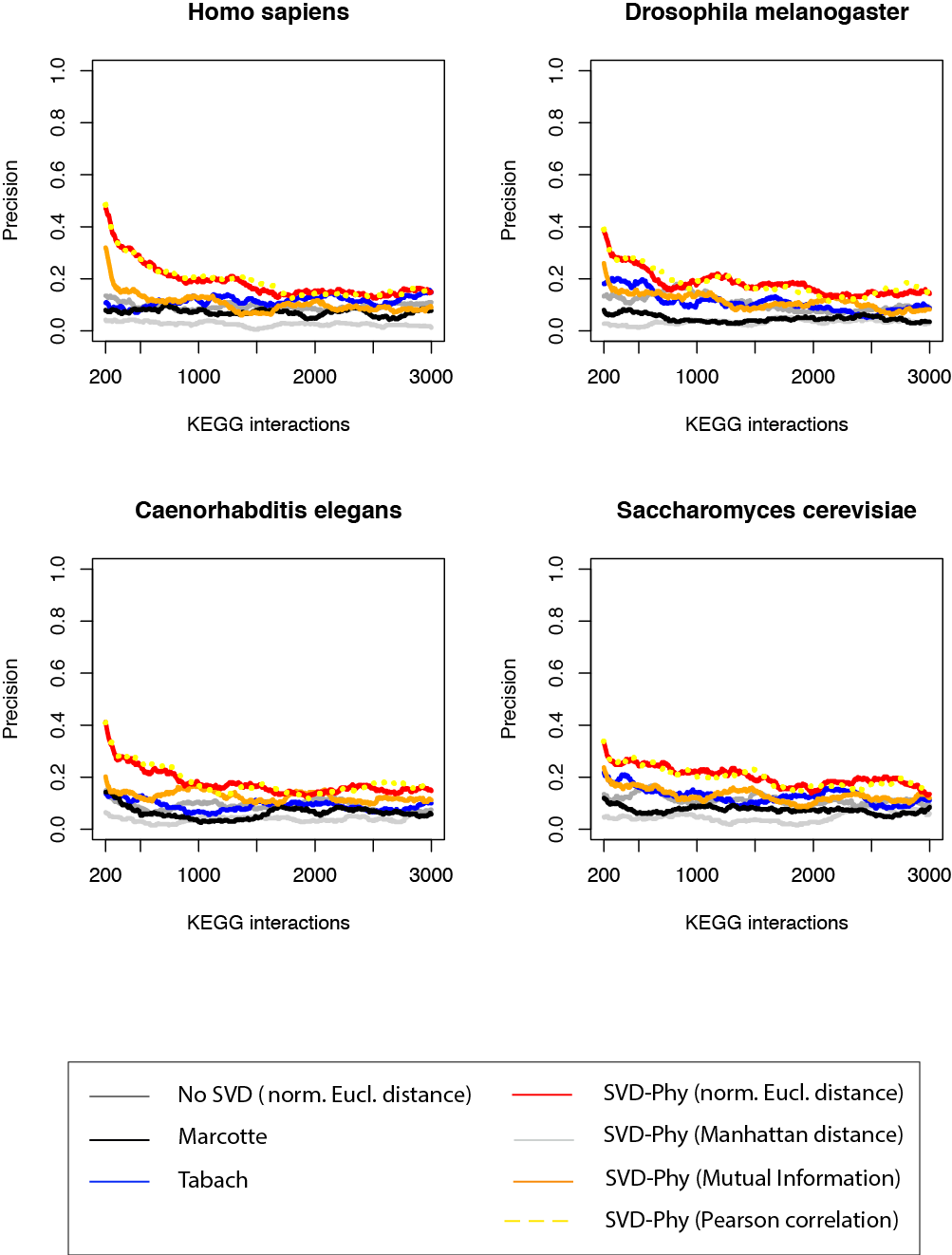


Figure S9. Benchmark of SVD-Phy, SVD-Phy using three alternative similarity metrics, SVD-Phy with the truncated SVD step disabled, the Marcotte algorithm (Date and Marcotte, 2003) and the Tabach algorithm (Tabach et al., 2013). Graphs show the precision [TP/(TP+FP)] which we estimated scanning the sorted lists with a sliding window of 400 interactions. We ran the algorithms using profiles composed of all 238 eukaryotes in STRING v10 (table S2). We removed from KEGG the uninformative maps (table S1). In SVD-Phy we retained the following percentages of columns from the rectangular SVD matrix U: *H. sapiens* 35%, *D. melanogaster* 15%, *C. elegans* 15%, and *S. cerevisiae* 15%.


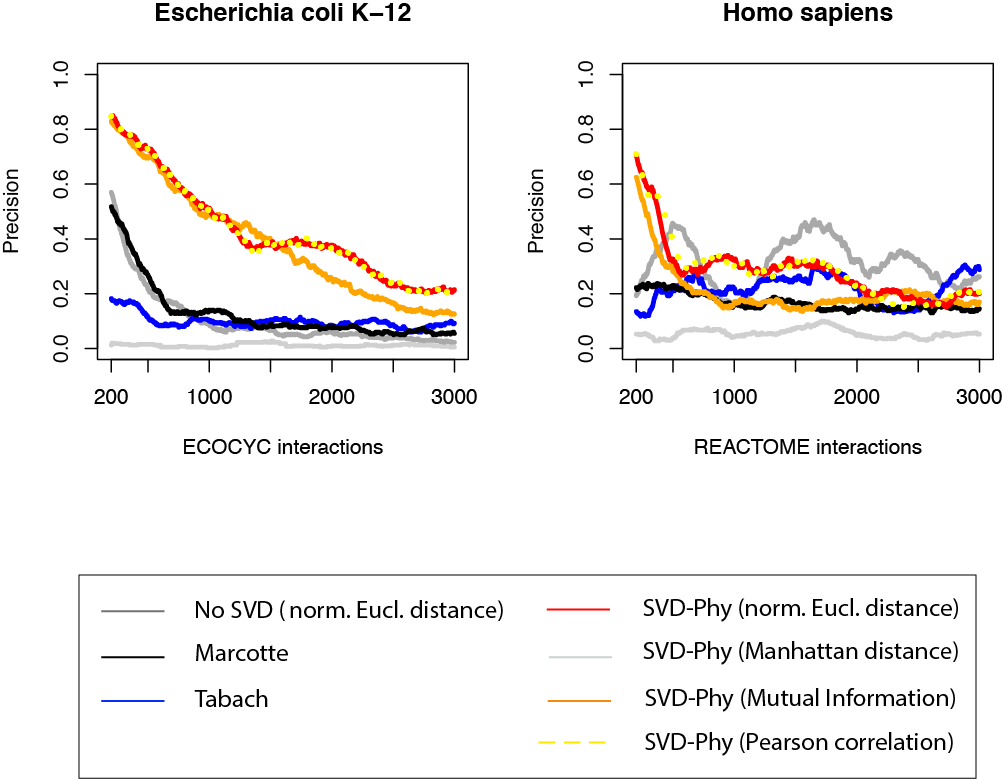


Figure S10. Benchmark results against EcoCyc and Reactome. Using the same methodology as in the KEGG benchmarks (figures S6–S9), we evaluated the predicted associations for *E.coli* and *H. sapiens* against EcoCyc and Reactome pathways, respectively. This shows that the using of different benchmark sets leads to the same conclusions regarding which methods and similarity metrics perform best. In SVD-Phy, we retained the same percentages of columns from the rectangular SVD matrix U as in the KEGG benchmarks, namely 15% and 35% for *E. coli* and *H. sapiens* respectively.


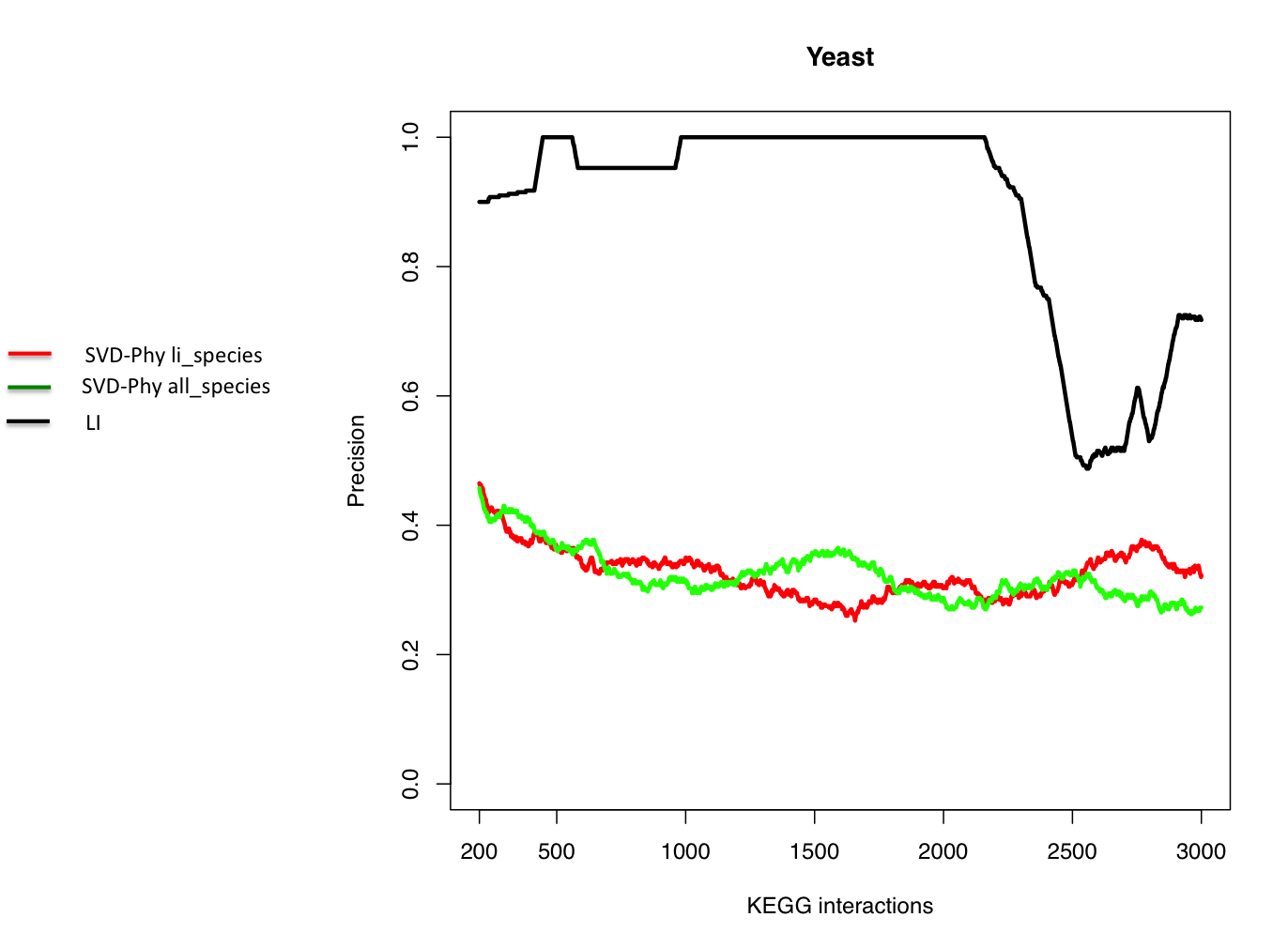

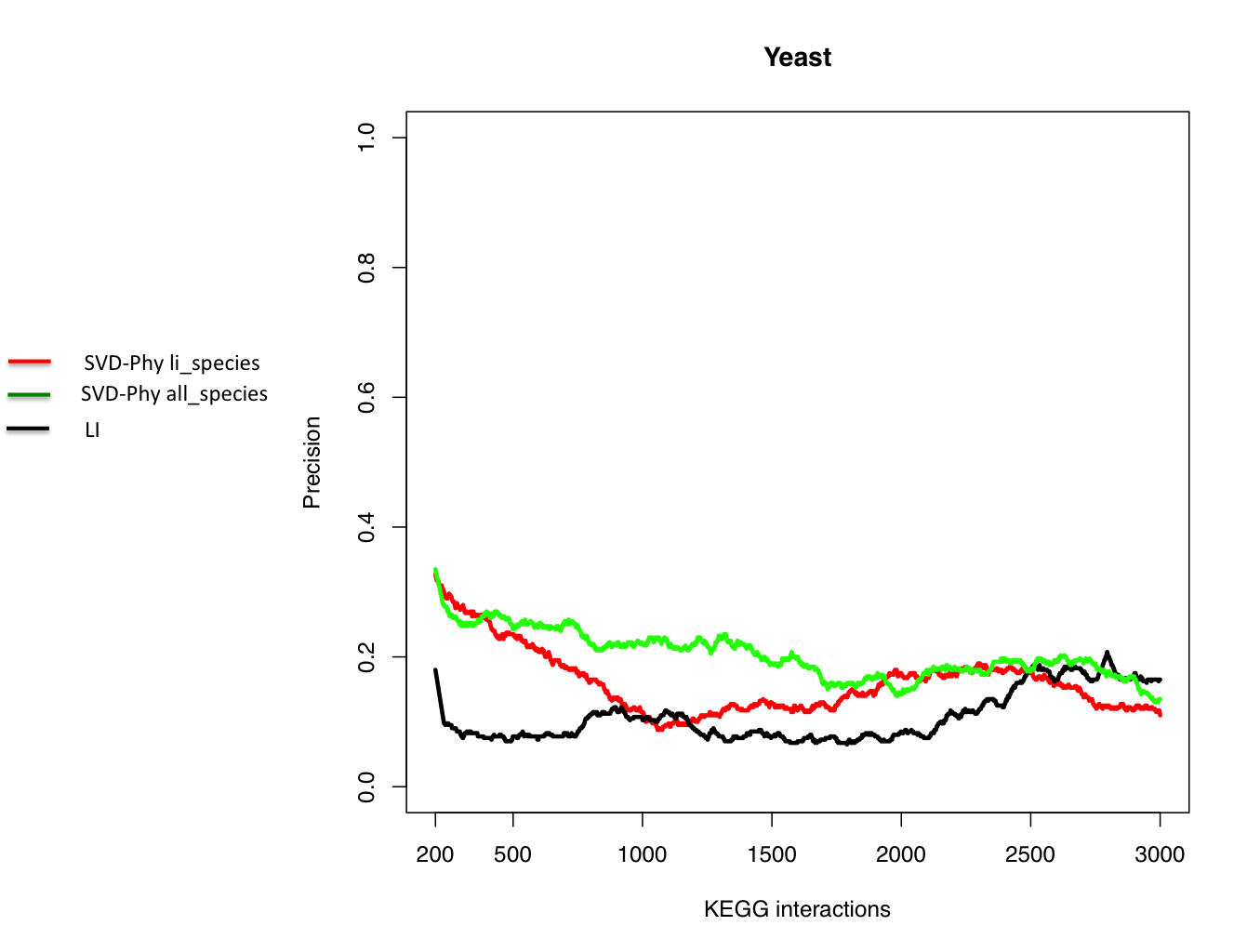


Figure S11. Comparison of SVD-Phy and CLIME. Unlike SVD-Phy, CLIME is computationally extremely intensive and thus not computationally tractable to run for the human genome (Li et al., 2014). To compare the methods, we thus opted to download the predictions for *S. cerevisiae* from the CLIME website (black) and compare them to SVD-Phy using profiles consisting of either all 238 eukaryotes (green) or only the 129 eukaryotes also used by CLIME (red). Graphs show the precision [TP/(TP+FP)], which we estimated by scanning the sorted lists with a sliding window of 400 interactions. For these benchmarks we either used all maps in KEGG (left) or left out the uninformative maps listed in table S1 (right). As can be seen, leaving out the uninformative KEGG maps makes a huge difference to the estimated performance of CLIME. We thus conclude that the seemingly exceptional performance of CLIME is almost entirely due to the method predicting a very large number of interactions within a small number of large KEGG maps. Specifically, we found that these many predictions fall primarily within a handful of very large KEGG maps that represent broad functions, namely “Ribosome”, “Biosynthesis of Amino Acids”, “Metabolic pathways”, “Metabolic metabolism in diverse environments” and “Biosynthesis of secondary metabolites”. In our opinion it is not desirable to link, for example, proteins involved in biosynthesis of unrelated amino acids or secondary metabolites to each other. As is evident, such maps can completely skew benchmark results, which is why is why we benchmarked all methods both with and without them.


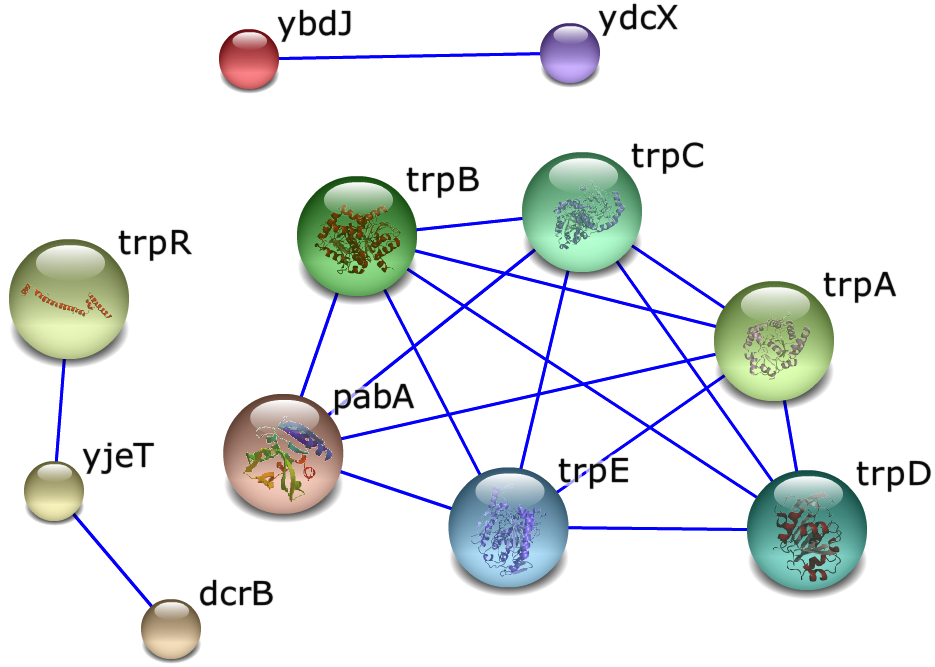


Figure S12. A predicted protein association network from SVD-Phy visualized in the STRING network viewer. The interactions of trpA with trpB and trpE are correctly inferred by SVD-Phy but not by the other methods tested (threshold set at a precision of 0.5).


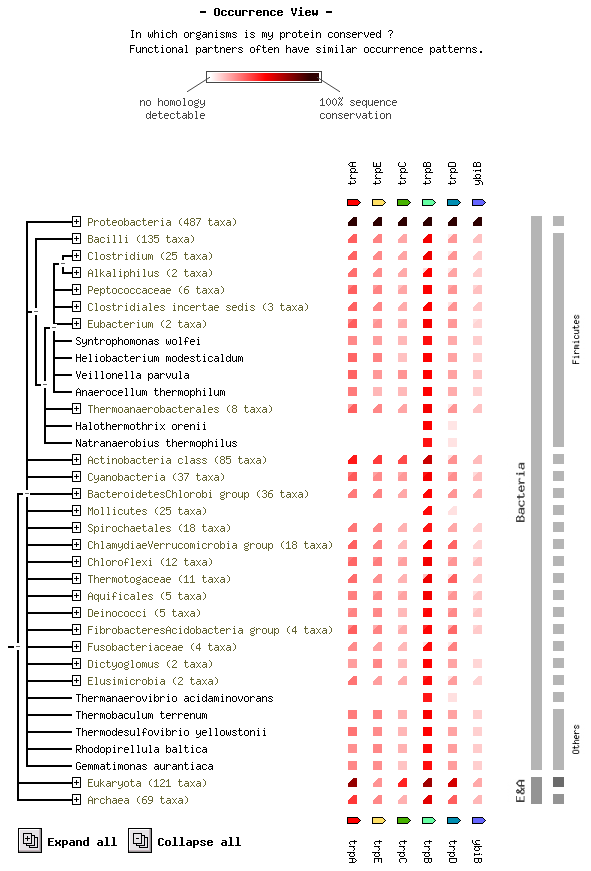


Figure S13. Visualization of phylogenetic profiles by the STRING evidence viewer. Proteins are listed across the top of the page and a phylogenetic tree with species names is listed below on the left hand side. In the subsequent grid, the presence of the protein in a species is marked with a red square and absence with a white space. The red color intensity of the square reflects the amount of sequence conservation of the homologous protein in the species. The tree can be expanded at various points; the upper left corner on the colored square indicates whether the protein is absent in at least one organism in the subtree.
